# High pollutant exposure level of the largest European community of bottlenose dolphins in the English Channel

**DOI:** 10.1038/s41598-019-48485-7

**Published:** 2019-09-12

**Authors:** Cyrielle Zanuttini, François Gally, Georges Scholl, Jean-Pierre Thomé, Gauthier Eppe, Krishna Das

**Affiliations:** 1 Groupe d’Etude des Cétacés du Cotentin (GECC), Place des Justes, 50130 Cherbourg-Octeville, France; 2 CART, UR MolSys B6c, University of Liège, 4000 Liège, Belgium; 3 CART-LEAE, Freshwater and Oceanic sciences Unit of reSearch (FOCUS- CART-LEAE), B6C, University of Liège, Liège, Belgium; 4 Freshwater and Oceanic sciences Unit of reSearch (FOCUS-Oceanology), B6C, University of Liège, Liège, Belgium

**Keywords:** Conservation biology, Marine biology

## Abstract

The objective of this study was to assess the levels of persistent organic pollutants (POPs) and mercury (T-Hg) in the blubber and skin, respectively, of the free-ranging bottlenose dolphins, *Tursiops truncatus*, from the Normanno-Breton Gulf, one of the largest identified coastal population in Europe. Among all the POPs analysed in this study, the ∑NDL-PCBs were the most abundant compounds found in the blubber (mean: 1.33 × 10^5^–0.65 × 10^5^ ng.g^−1^ lipid weight (lw) for males and females respectively), followed by ∑DDX (1.11 × 10^4^–4.67 × 10^3^ ng.g^−1^ lw) > ∑DL-PCBs (8.06 × 10^3^–2.62 × 10^3^ng.g^−1^ lw) > ∑PBDEs (1.95 × 10^3^–0.64 × 10^3^ng.g^−1^ lw) > dieldrin (1.86 × 10^3^–0.18 × 10^3^ ng.g^−1^ lw) > ∑endosulfan (405–62 ng.g^−1^ lw) > HCB (86–52 ng.g^−1^ lw) > ∑HCHs (47–60 ng.g^−1^ lw) > ∑chlordane (24–0.97 ng.g^−1^ lw) > ∑PCDFs (0.3–0.1 ng.g^−1^ lw) > ∑PCDDs (0.06–0.05 ng.g^−1^ lw). The T-Hg concentrations were highly variable between individuals (2.45 × 10^3^ ng.g^−1^ to 21.3 × 10^3^ ng.g^−1^ dry weight, dw). The reported concentrations are among the highest reported for cetaceans. We strongly recommend that the Normanno-Breton Gulf be a special area of conservation (cSAC) candidate because it contains the last large European population of bottlenose dolphins (rare or threatened within a European context) designated under the EC Habitats Directive.

## Introduction

Most organochlorinated contaminants were banned in developed countries in the 1970s and 1980s. However, their persistent chemical properties favour their long-range transport and remanence in water, air and biota, including the deepest ocean fauna^1^. Polychlorinated biphenyls (PCBs) are persistent organic pollutants (POPs) that pose a serious environmental threat to wildlife and humans^2,3^. Very recently, a meta-analysis of stranded and biopsied cetaceans in Europe stressed that the bottlenose dolphin, *Tursiops truncatus*, had markedly elevated PCB concentrations in blubber^4^. In particular, the “PCB hotspots” included the Strait of Gibraltar, south western Iberia, the Gulf of Cadiz and the Mediterranean Sea. These high PCB concentrations are still a major cause of decline in European cetacean populations^2^.

The bottlenose dolphin is a relevant species for examining environmental contamination trends in coastal areas^5,6^: The species is widely distributed in estuarine and nearshore waters^7^, is a long-lived species with a high trophic position in the marine food web and a thick layer of blubber in which lipophilic pollutants accumulate^8^. Previous risk assessment investigations on the potential effect of PCB exposure demonstrated their likely effects on offspring survival in bottlenose dolphins^9^, especially reduced first-year survivorship of calves and a reduced annual population growth^10,11^.

**Figure 1:**
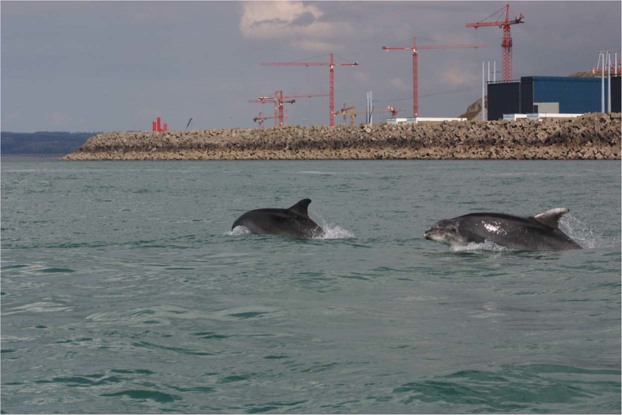
The bottlenose dolphin *Tursiops truncatus* in the Normanno-Breton Gulf (English Channel). Photo Credit: GECC.

Approximately 420 coastal bottlenose dolphins inhabit the Normanno-Breton Gulf (NBG) in the English Channel^12^ (Fig. 1).

The size of coastal communities of bottlenose dolphins in European waters ranges from approximately 10 to 300 individuals^13–18^. Therefore, the coastal population of bottlenose dolphins from the NBG is one of the largest identified among dolphin communities in Europe^12^. Previous studies have shown an interannual site fidelity to the NBG and that this population is genetically isolated from neighbouring populations living off the United Kingdom and Ireland^12^. The bottlenose dolphins of the NBG are a community that remains poorly known. To date, no toxicological data are available on the bottlenose dolphins from the NBG despite their proximity to urban and industrial activities. Quantifying the baseline concentrations and patterns of POPs and total mercury (T-Hg) in bottlenose dolphin populations is critical for risk assessment and long-term management. These organic chemical substances are classified as persistent organic pollutants by the Stockholm Convention (http://www.pops.int) because of their toxicity, lipophilic properties and widespread global distribution. The Minamata Convention (http://www.mercuryconvention.org/Home/tabid/3360/language/en-US/Default.aspx) on Mercury drew attention to this toxic metal that is released to the atmosphere, water and soil from a variety of anthropogenic sources, which leads to its bioaccumulation in biota.

Biopsy samples of skin and blubber were collected from 82 bottlenose dolphins from the NBG in the English Channel to assess the concentrations of a large range of industrial compounds, dioxin-related compounds (DRCs) and organochlorinated pesticides: NDL-PCBs and DL-PCBs (non-dioxin-like polychlorinated biphenyls and dioxin-like polychlorinated biphenyls, respectively), PBDEs (polybrominated diphenyl ethers), DDXs (dichloro-diphenyl-trichloroethane and metabolites), HCHs (hexachlorocyclohexanes), HCB (hexachlorobenzene), PCDDs (polychlorinated dibenzo*-p-*dioxins), PCDFs (polychlorinated dibenzofurans), cis-chlordane, trans-chlordane, α-endosulfan, β-endosulfan, endosulfan-sulfate, and dieldrin, in the blubber. T-Hg (total mercury) was analysed in the skin.

## Results

The lipid content in the blubber ranged from 2.4 to 22.8% (Table 1). No differences were observed between the sex classes, age classes and sampling season. The bottlenose dolphins sampled in 2010 showed a slightly higher lipid percentage than those sampled in 2012 (13.6% and 8.5%, respectively; Kruskal-Wallis: *p* = 0.0477).

**Table 1:** Lipid percentage (%) and POP concentrations (ng.g^−1^ lw) in the blubber biopsies of the bottlenose dolphins, *Tursiops truncatus*, from the Normanno-Breton Gulf.

	Males	Females	*p*-value
Lipids %	11 (11) ± 6	11 (11) ± 4	0.831
	(2–23)	(3–17)	
	n = 47	n = 11	
Σ6 NDL-PCBs^a^	1.33 × 10^5^ (1.14 × 10^5^) ± 7.89 × 10^4^	6.45 × 10^4^ (5.14 × 10^4^) ± 7.41 × 10^4^	**0.001**
	(1.75 × 10^4^–3.93 × 10^5^)	(4.5 × 10^3^–2.7 × 10^5^)	
	n = 47	n = 11	
ΣPBDEs^b^	1.95 × 10^3^ (1.78 × 10^3^) ± 1.07 × 10^3^	639 (214) ± 686	**0.0001**
	(195–3.87 × 10^3^)	(51–2.14 × 10^3^)	
	n = 47	n = 11	
p,p’DDE	1.04 × 10^4^ (8.07 × 10^3^) ± 1.05 × 10^4^	4.19 × 10^3^ (3.58 × 10^3^) ± 3.79 × 10^3^	**0.0006**
	(1.43 × 10^3^–7.17 × 10^4^)	(302–1.45 × 10^4^)	
	n = 47	n = 11	
p,p’DDD	279 (275) ± 163	147 (138) ± 150	**0.021**
	(1 − 703)	(1 − 455)	
	n = 47	n = 11	
p,p’DDT	149 (71) ± 201	104 (7) ± 144	0.578
	(6–940)	(6–434)	
	n = 47	n = 11	
ΣDDXs^c^	1.11 × 10^4^ (8.39 × 10^3^) ± 1.08 × 10^4^	4.67 × 10^3^ (4.33 × 10^3^) ± 4.01 × 10^3^	**0.0008**
	(1.64 × 10^3^–7.43 × 10^4^)	(362–1.54 × 10^4^)	
	n = 47	n = 11	
ΣHCHs^d^	46.5 (34) ± 41.2	60 (28) ± 73.5	**0.968**
	(7–229)	(12–258)	
	n = 47	n = 11	
HCB	85.6 (80.7) ± 43.6	52 (50) ± 42	**0.034**
	(18–271)	(6.5–120)	
	n = 47	n = 11	
ΣChlordane	25 (15) ± 28	0.97 (1.04) ± 0.2	**<0.0001**
	(3.9–99)	(0.74–1.1)	
	n = 18	n = 3	
Dieldrin	1.86 × 10^3^ (1.62 × 10^3^) ± 1.23 × 10^3^	180 (137) ± 147	**0.002**
	(615–5.38 × 10^3^)	(59–344)	
	n = 18	n = 3	
ΣEndosulfan	405 (350) ± 214	62 (77) ± 44	**<0.0001**
	(181–873)	(13–98)	
	n = 18	n = 3	

The data are presented as the mean (median) ± the standard deviation (min–max values) and the number of individuals (n). The differences between the males and females are shown as the p-values from the Mann-Whitney test. Significant p-values are in bold.

^a^∑ 6 NDL-PCBs: CB 28, CB 52, CB 101, CB 138, CB 153, and CB 180.

^b^∑ PBDEs: BDE 28, BDE 47, BDE 66, BDE 85, BDE 99, BDE 100, BDE 153, BDE 154 and BDE 183.

^c^∑ DDXs: *o*,*p’*-DDT, p,p’-DDE, *p*,*p’*-DDD, *p*,*p’*-DDT, *o*,*p’*DDE, and *o*,*p’*DDD.

^d^∑ HCH: α-HCH, β-HCH, and γ-HCH.

All the measured compounds were detected at quantifiable levels (>limit of quantification (LOQ)) with the exception of endosulfan-α (below the LOQ), β-HCH (detected in 5 out of 58 samples), BDE 66 (detected in 7 out of 58 samples), three dioxins (below the LOQ: 2, 3, 7, 8-TetraCDD; 1, 2, 3, 7, 8-PentaCDD; and 1, 2, 3, 4, 7, 8-HexaCDD) and one furan (detected in 1 out of 12 samples: 1, 2, 3, 4, 7, 8, 9-HeptaCDF).

Among all the persistent organic pollutants analysed in this study (Tables 1 and 2 and Table S2, Supplementary Information), the ∑NDL-PCBs were the most abundant compounds found in the blubber, followed by ∑DDX > ∑DL-PCBs > ∑PBDEs > dieldrin > ∑endosulfan > HCB > ∑HCHs > ∑chlordane > ∑PCDFs > ∑PCDDs.

**Table 2:** Dioxin-related compounds (DRC, pg.g^−1^ lw and pg WHO-TEQ.g^−1^ lw) in the blubber biopsies of bottlenose dolphins from the Normanno-Breton Gulf.

	Males n = 9		Females n = 3	
	pg.g^−1^ lw	WHO-TEQ	pg.g^−1^ lw	WHO-TEQ
ΣPCDDs	60 ± 36	1 ± 0.45	51.5 ± 23.5	0.96 ± 0.4
	(32–149)	(0.7–2)	(30–77)	(0.53–1.3)
ΣPCDFs	282 ± 92	26 ± 8.7	102 ± 20	7.7 ± 0.7
	(140–429)	(12–401)	(79–117)	(7–8.4)
Σ non-ortho PCBs	4.41 × 10^3^ ± 3.2 × 10^3^	183 ± 98	3.55 × 10^3^ ± 1.62 × 10^3^	84 ± 33
	(1.94 × 10^3^–1.23 × 10^4^)	(95–417)	(1.68 × 10^3^–4.54 × 10^3^)	(47–109)
Σ mono-ortho PCBs	8.05 × 10^6^ ± 9.41 × 10^5^	242 ± 28	2.61 × 10^6^ ± 2.17 × 10^6^	78 ± 65
	(7.23 × 10^6–9.65^ × 10^6^)	(217–290)	(1.1 × 10^6^–5.1 × 10^6^)	(33–153)
Σ DRCs	8.06 × 10^6^ ± 9.4 × 10^5^	451 ± 93	2.62 × 10^6^ ± 2.16 × 10^6^	171 ± 92
	(7.23 × 10^6^–9.66 × 10^6^)	(370–668)	(1.12 × 10^6^–5.1 × 10^6^)	(88–270)

The data are presented as the mean ± the standard deviation (min–max values) and the number of individuals (n).

### Polychlorinated biphenyls (PCBs)

The ∑6 NDL-PCBs were the major contaminant groups found in the blubber of all the dolphins, with the contribution of the total POPs measured as 91% and 92% for the males and females, respectively. The ∑6 NDL-PCB concentrations were significantly higher in the males than in the females (Mann-Whitney: w = 103, *p* = 0.0014). The adult males had significantly higher ∑6 NDL-PCBs compared to the adult females and sub-adults (Kruskal-Wallis: X^2^ = 6.98, *p* = 0.0225; X^2^ = 11.94, *p* = 0.0016, respectively). No significant difference was found between the adult females and sub-adults (Kruskal Wallis: X^2^ = 0.0002, *p* = 0.9991).

**Figure 2:**
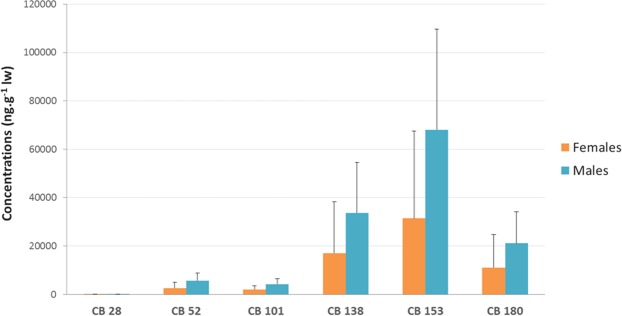
Mean concentrations (and standard deviation) of Σ6 NDL-PCBs (ng.g^−1^ lw) in blubber biopsies of bottlenose dolphins from Normanno-Breton Gulf.

Among the 6 NDL-PCBs, CB 153 was the predominant congener found in the blubber, with contributions of 51% and 49% of the ∑6 NDL-PCBs in the males and females, respectively (Fig. 2), followed by CB 138 (25% and 26% in the males and females, respectively) and CB 180 (16% and 17% in the males and females, respectively). With the exception of CB 28, all 6 NDL-PCB congeners were significantly higher in the males than in the females (Mann-Whitney, *p* < 0.05).

### Dioxin-related compounds (DRCs)

The dioxin-like PCB (non-ortho and mono-ortho), PCDD and PCDF concentrations and their respective WHO-TEQ values (pg.g^−1^, lw) are presented in Tables 2 and S2 (Supplementary Information). The use of toxic equivalency factors (TEFs) to achieve toxic equivalency (TEQ) allows the assessment of the toxic potential of pollutants capable of triggering Aryl hydrocarbon (Ah) receptor-mediated effects, such as DRCs^19^. The toxic equivalency factor (TEF) expresses the toxicity of dioxins, furans and DL-PCBs in terms of the most toxic form of dioxin, 2, 3, 7, 8-TCDD^20^.

**Figure 3:**
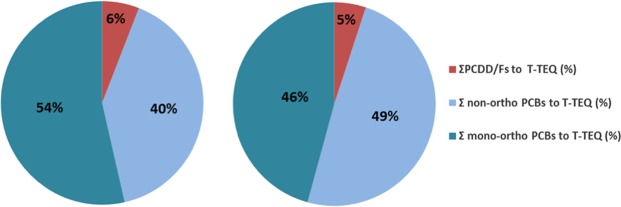
Mean proportions of PCDD/Fs and DL-PCBs (TEQ) in blubber biopsies of bottlenose dolphins from the Normanno-Breton Gulf in males (left) and females (right).

The DL-PCB concentrations represented only 5% of the total ∑PCB concentrations measured in this study (6 NDL-PCBs plus DL-PCBs) but were nevertheless the predominant groups of DRCs found in the blubber biopsies of the bottlenose dolphins from the NBG (Fig. 3). The PCDD/Fs only contributed 6% of the total TEQ in both the males and females of the population.

Among the DL-PCBs, PCB 126 and PCB 118 contributed to the majority of the total TEQ, with contributions of 36% and 31% in the males, respectively, and contributions of 45% and 23% in the females, respectively.

### Other compounds

The PBDE concentrations represented less than 2% of the total sum of the persistent organic pollutants measured in the blubber biopsies of the bottlenose dolphins from the NBG. BDE 47 was the most predominant congener found in the blubber, with a contribution of 60% of the total ∑PBDEs.

**Figure 4:**
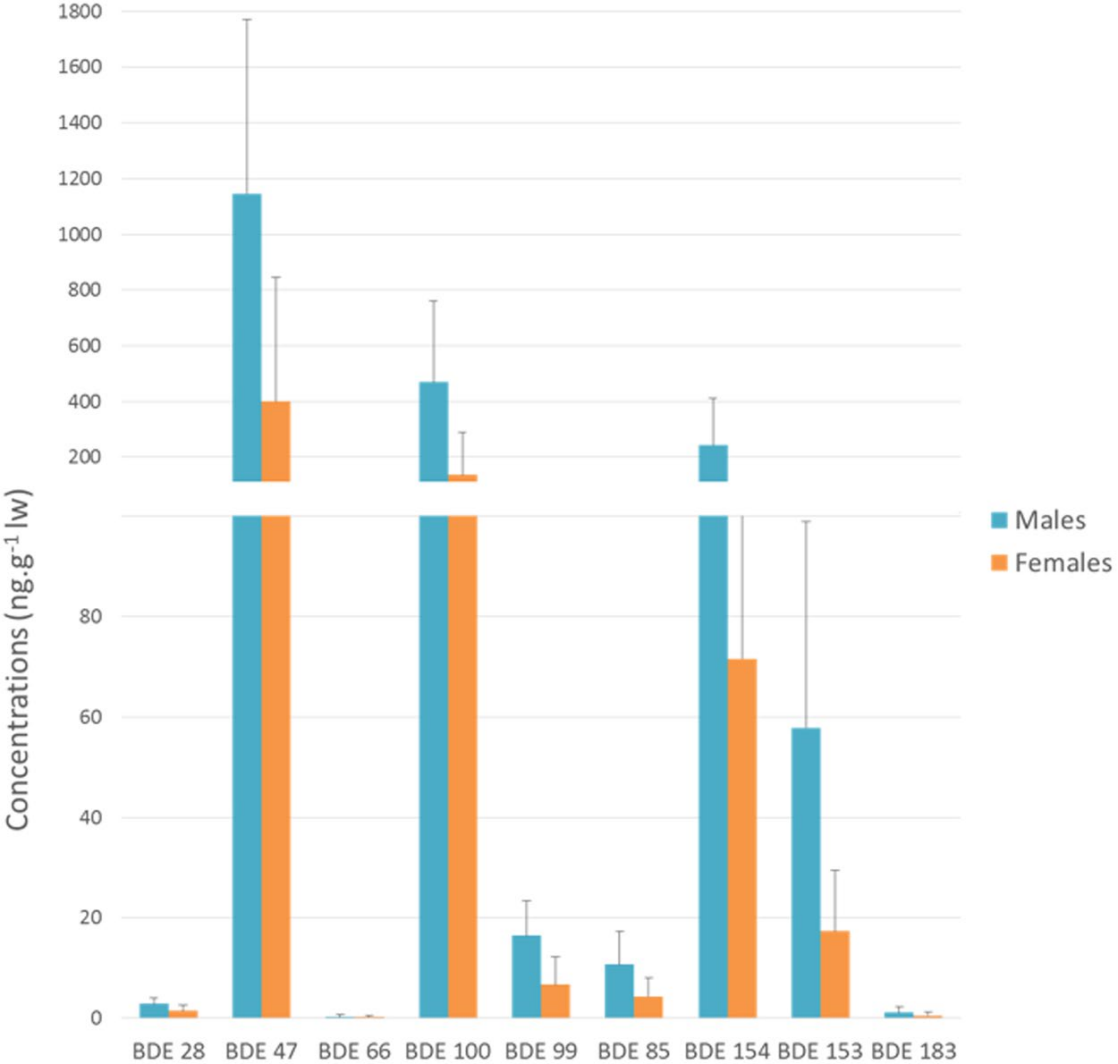
Mean concentrations (and standard deviations) of ƩPBDEs (ng.g^−1^ lw) in blubber biopsies of bottlenose dolphins from the Normanno-Breton Gulf.

The PBDE concentrations occurred in the following order: BDE 47 > BDE 100 > BDE 154 > BDE 153 > BDE 99 > BDE 85 > BDE 28 > BDE 183 > BDE 66 (Fig. 4).

The concentrations of the PBDE congeners were higher in the males than in the females (Mann-Whitney U Test, *p* = 0.0001). Our results suggest an age-dependent accumulation for the PBDEs, with adult males having greater concentrations than sub-adult dolphins (Kruskal-Wallis, X^2^ = 9.925, *p* = 0.007). The ∑PBDE concentrations were significantly lower in the adult females than in the sub-adults (Mann-Whitney U Test, *p* = 0.019). Compared to the adult males, the adult females showed a lower proportion of BDE 47 and a higher proportion of the highly brominated PBDEs, such as BDE 153 and 154.

The ΣDDX compounds were the predominant organochlorine pesticides found in the dolphin blubber, representing between 86% and 99% of the total pesticides. Significantly higher levels of ΣDDXs were detected in the males than in the females (Table 1). The adult males also had significantly greater ΣDDX concentrations than the adult females and sub-adults. The dieldrin and endosulfan levels were significantly higher in the males than in the females.

The total mercury (T-Hg) was detected at quantifiable levels in all of the skin biopsies of the bottlenose dolphins from Normanno-Breton Gulf. The T-Hg concentrations were highly variable between individuals, ranging from 2.45 × 10^3^ to 2.13 × 10^4^ ng.g^−1^ dw, with no significant difference between the males and females (Mann-Whitney test, p = 0.44, Table 3).

**Table 3:** Total mercury concentrations (ng.g^−1^ dry weight, dw) in the skin biopsies of the bottlenose dolphins from Normanno-Breton Gulf.

Males	Females
9.42 × 10^3^ (9.32 × 10^3^) ± 3.53 × 10^3^	1.12 × 10^4^ (9.24 × 10^3^) ± 5.69 × 10^3^
(2.45 × 10^3^–1.74 × 10^4^)	(3.03 × 10^3^–2.13 × 10^4^)
n = 49	n = 20

The data are presented as the mean (median) ± the standard deviation (min–max values) and the number of individuals analysed (n).

### Correlation between the stable isotopes and pollutants

The δ^13^C values in the skin were correlated with the blubber ∑PBDE concentrations (*p* = 0.024, R^2^ = 0.296). The δ^13^C and δ^15^N values were correlated with the T-Hg concentrations in the skin (*p* < 0.0001, R^2^ = 0.63 and *p* < 0.0001, R^2^ = 0.48 for δ^13^C and δ^15^N, respectively, (Fig. S1, Supplementary Information). There was no correlation between the stable isotopes and the other POP concentrations.

## Discussion

**Figure 5:**
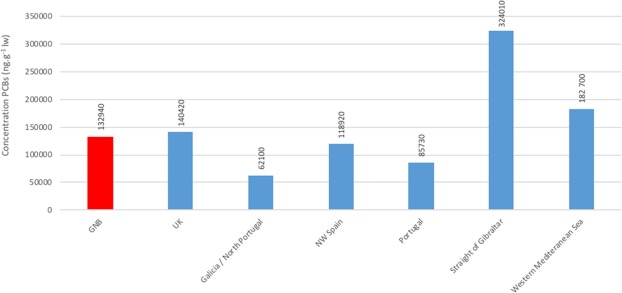
Mean PCB concentrations in male bottlenose dolphins from the Normanno-Breton Gulf (NBG, red bar, present study, Σ6 NDL-PCBs) compared to the ΣPCBs from other European locations^4^.

The bottlenose dolphins from the NBG have markedly elevated blubber NDL-PCB concentrations compared to the other concentrations previously described in bottlenose dolphins from European waters^4^ (Fig. 5).

The NDL-PCBs accounted for more than 91% of all the analysed organic compounds, with concentrations exceeding 393000 ng.g^−1^ lw in one male dolphin.

Among the NDL-PCBs, the hexachlorobiphenyls (PCB 153 and PCB 138) were the major compounds (ranging from 64 to 80%), followed by the heptachlorobiphenyls (PCB 180). PCB 153, 138 and 180 were the dominant PCBs detected in the marine mammals due to their widespread use, persistence, high lipophilicity and structural resistance to metabolism^21–24^. This pattern is very similar to that found by the Seine-Normandy Water Agency in marine biota from the English Channel, with hexachlorobiphenyls representing more than 60% of the NDL-PCB concentrations^25^.

The PCBs were used mainly as dielectric fluids in industrial capacitors and transformers^26^. The commercialization and use of PCBs have been prohibited in France since 1987^27^. The Seine is a hotspot for PCBs^28,29^, and the NBG, which is close to the Seine estuary, is an environment with high industrial, agricultural and urban activities. Indeed, several studies (including those by the International Pellet Watch, Minier and co-authors^28^) the pollution monitoring conducted by the Seine-Normandy Water Agency and the monitoring network (ROCCH) on mussels and oysters along the French coastline (http://www.ifremer.fr), have shown that the Seine is one of the most polluted rivers in the world for PCBs.

Recently, toxicology thresholds have been used to evaluate the actual impact of PCBs on porpoise and dolphin populations in European waters^4,30^. A lower PCB toxicity threshold of 17 mg.kg^−1^ lw (as Aroclor 1254) was used for the onset of reproductive impairment and immune suppression in marine mammals^31^ and was calculated to be equivalent to 9.0 mg.kg^−1^ lw (as ΣPCB)^4^. A higher PCB toxicity threshold of 77 mg.kg^−1^ lw (as Clophen 50), which causes reproductive failure in Baltic ringed seals^32^, was calculated to be equivalent to 41 mg.kg^−1^ lw (as ΣPCB)^4^. 57 bottlenose dolphins (out of 58) exceeded the lower thresholds, and 51 bottlenose dolphins (out of 58) exceeded this higher threshold.

The toxicity threshold was exceeded in 50% of the common dolphins sampled in the waters off the French coast^33^. The threshold was exceeded the least in dolphins (9%) and porpoises (25%) off the coast of Ireland. The authors reported an arithmetic mean PCB concentration of 1.37 × 10^4^ ng.g^−1^ lw in 36 female common dolphins from the French coast of the English Channel and the Atlantic, which is 4 times lower than the concentration measured in the female bottlenose dolphins from the NBG. The high values observed in the bottlenose dolphins from the NBG reflect their higher exposure, which is linked to their coastal habitat, feeding, and larger body size.

The male bottlenose dolphins displayed higher NDL-PCB concentrations than the females. These results are consistent with the general pattern previously reported in which females offloaded a large proportion of PCBs to their young during gestation and more specifically during lactation, while males continue to bioaccumulate PCBs throughout life^34,35^. The PCB accumulation in a bottlenose dolphin population off Sarasota (US) was associated with a reduced annual growth rate of 3.6%^11^. A recent study linked the decline of the population of killer wales in Europe with their PCB levels, which were likely responsible for reproductive failure^2^. Our results indicated the important transfer of PCBs by females to their young, which may raise concern for the population. Indeed, the immune system and metabolic capacities are less developed in foetuses and juveniles, making them more sensitive to the adverse effects of PCBs^36^. The transfer of PCBs and other organohalogenated compounds is a well-described process in cetaceans. Female harbour porpoises pass approximately 50% of the PCB 153 levels in their blubber on to their offspring. The mother is assumed to provide the same amount of milk and nutrients during each single cycle. Therefore, the concentrations of PCBs will decrease. As a result, the firstborn will be more contaminated than the last born. High PCB contamination of the calves combined with their insufficient metabolism at younger ages leaves this group vulnerable^36^.

The NDL-PCBs were the most abundant compounds in terms of their concentrations, but they were clearly not the only ones: other toxic organohalogenated compounds were detected in the blubber of bottlenose dolphins from the NBG in the following order: ∑DDX > ∑DL-PCBs > ∑PBDEs > dieldrin > ∑endosulfan > HCB > ∑HCHs > ∑chlordane > ∑PCDFs > ∑PCDDs. The combined effects of this cocktail of pollutants may be more complex than that anticipated using toxicity models.

Similar to the PCBs, the female bottlenose dolphins from the NBG displayed lower concentrations of flame retardants (PBDEs), organochlorinated pesticides (DDX and pp’DDE, HCB, chlordane, dieldrin and endosulfan) and some DRC congeners (PCDFs and mon-ortho PCBs), meaning that all these compounds (1) can reach very high concentrations in older male bottlenose dolphin and that (2) female dolphins offload a significant portion of this cocktail to their offspring during gestation and lactation, placing foetuses and newborns at a higher risk.

DRCs are produced by various processes such as the synthesis of some pesticides and PCBs and paper bleaching as well as the burning of vegetation and waste^37^. DRCs exert their toxic effects through their interaction with the Ah receptor, an intracellular protein^38^. Exposure to DRCs has been associated with a multitude of adverse health effects. Reproductive system and immunotoxicity effects appear to be among the most sensitive responses^38^. The TEQ values result from the concentrations of the different DRCs as well as from the ability of each compound to induce the Ah-receptor-mediated response^39^.

DL-PCBs in the bottlenose dolphins from the NBG represented the most important contribution (94%) for the toxicity equivalences of the DRCs (expressed as the TEQ). Our results were similar to the results found for Guiana dolphins (*Sotalia guianensis*) from the highly contaminated Guanabara Bay in Brazil, where the DL-PCBs represented 98.8% of the total TEQ^40^. The DL-PCBs represented 81% and 65% of the total TEQs analysed in the bottlenose dolphins from the lower Florida Keys and the Florida coastal Everglades, respectively^6^.

To our knowledge, only one threshold for the immunotoxic effects of DRCs exists for cetaceans, at 255 pg.g^−1^ WHO-TEQ lw^41^.

The total TEQ in the NBG bottlenose dolphins samples is easily comparable to the adverse effect levels established for killer whales^41^, suggesting that the majority of free-ranging bottlenose dolphins in the NBG are at risk for the toxic effects associated with DRCs.

The mercury concentrations in the skin from the NBG bottlenose dolphins are among the highest concentrations observed in this species (Table S3, Supplementary Information) and are very close to the concentrations previously described for the bottlenose dolphins in the Mediterranean Sea^42^ and from the Florida coastal Everglades^6^, two sites that are known for their high Hg contamination levels from both anthropogenic and natural origins. Our data suggest a high exposure of the NBG bottlenose dolphins linked to their high trophic position (Fig. S1) and their diet, which is composed of mackerel^43^.

The bottlenose dolphins in European waters are protected by the Habitats Directive (92/43/22C). Their conservation requires the creation of special areas of conservation (Annex II) and the need for strict protection (Annex IV).

Despite this European directive, human activities are increasing in the Normanno-Breton Gulf; the potential threats include pollutants, noise pollution, particularly construction noise, disturbance by tourism activities and bycatch^43^. Bottlenose dolphins have declined in the northeast Atlantic^44^. The historic stranding data in Europe suggest that coastal bottlenose dolphins became locally extinct or depleted in the late 1960s to the mid-1970s, including those in the UK^45^ and the Dutch coast^46^. The last member of a resident bottlenose dolphin population in Arcachon, France, died in 2001^47^, and the small resident bottlenose dolphins group in Portugal (Sado Estuary) declined over several decades due to low calf survival over several decades^15^.

As recently emphasized by Jepson and Law^3^, *there is an urgent need to review the methods of PCB mitigation in the marine environment in Europe*; in compliance with the Stockholm Convention, the goal is to drastically reduce the PCB input of the marine environment by 2028. PCB mitigation measures include *i*.*e*. a destruction of PCB stocks and PCB-containing equipment/buildings, and a limitation of PCB mobilization in marine sediments^4^.

We strongly recommend the Normanno-Breton Gulf as a special area of conservation (cSAC) candidate because it contains the last large European population of bottlenose dolphins (rare or threatened within a European context) designated under the EC Habitats Directive.

## Methods

A detailed methodology can be found in the supporting information.

### Specimens

**Figure 6:**
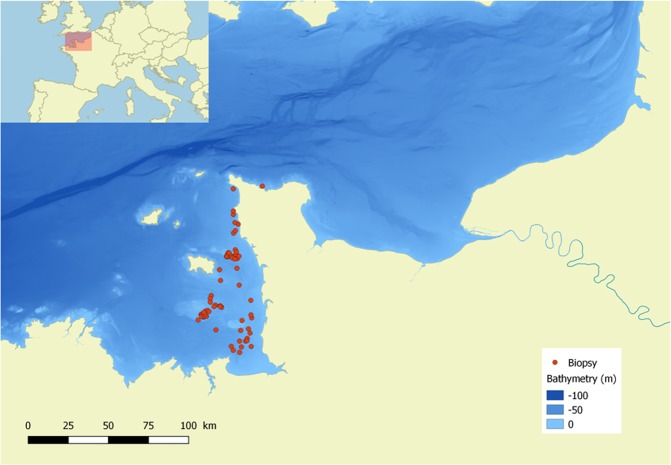
Sampling location of bottlenose dolphins *Tursiops truncatus* in the Normanno-Breton Gulf (English Channel).

Biopsy samples from individual bottlenose dolphins were collected during boat surveys in the Normanno-Breton Gulf from 2010 to 2012 (French ministry permit No. 09/115/DEROG) (Fig. 6).

From a total of 82 bottlenose dolphins biopsied, we obtained 79 blubber biopsies and 69 skin biopsies (Table S1, Supporting Information). The sex of each individual biopsy was determined previously^43^.

### Data presentation

The Σ 6 NDL-PCB congeners (28, 52, 101, 138, 153 and 180) were chosen as the priority compounds for the POP analysis by the Scientific Panel on Contaminants in the Food Chain of EFSA (CONTAM Panel)^48^. The ΣPBDEs is the sum of the 9 BDE congeners (28, 47, 66, 85, 99, 100, 153, 154 and 183); the ΣHCH is the sum of α-HCH, β-HCH and γ-HCH; the ΣDDXs is the sum of p,p’DDT, o,p’-DDT, p,p’-DDE, o,p’DDE, p,p’-DDD and o,p’DDD. For the stable isotope analysis, the δ^13^C and δ^15^N values analysed previously^43^ were integrated into the present manuscript.

### Persistent organic pollutant (POPs) and total mercury (T-Hg)

Blubber samples were analysed for non-dioxin-like (NDL) PCB congeners (28, 52, 101, 138, 153 and 180), dioxin-like (DL) PCB congeners (77, 81, 105, 114, 118, 123, 126, 156, 157, 167, 169 and 189), 17 WHO PCDD/Fs, PBDEs (28, 47, 66, 85, 99, 100, 153, 154 and 183) and organochlorinated pesticides (ΣDDXs, cis-chlordane and trans-chlordane, α-Endosulfan, β-Endosulfan and Endosulfan-sulfate, dieldrin, HCB, α-HCH, β-HCH, γ -HCH and HCH (See Supporting Information Table S1). The quantification of the PCDD/Fs, DL-PCBs, NDL-PCBs, PBDEs, DDXs, HCB and HCHs was performed by the isotope dilution technique using ^13^C labelled analogues^6,49^. Twenty-one blubber biopsies were selected for the analysis of the chlorinated pesticides cis-chlordane, trans-chlordane, α-endosulfan, β-endosulfan, endosulfan-sulfate, and dieldrin. The total mercury analysis was performed on the skin samples from 69 bottlenose dolphins, as was previously described^6^.

### Statistical analysis

Non-parametric statistics were used because the assumptions of normality (Shapiro test) and homoscedasticity (Bartlett test) of the data were not met, even after a log-transformation. Significant differences between the contaminant concentrations in the sex and marking levels were assessed using the Mann-Whitney *U* test for the comparison of the two categories, and the Kruskal-Wallis test was used when there were more than two categories. If a significant difference was found, a post hoc Nemenyi’s test was performed to identify which group was significantly different. Spearman rank correlation tests were performed to examine the potential linear associations between the contaminants and stable isotopes. A statistical significance level of 0.05 was applied for all the tests. The statistical analyses were conducted with R studio software (version 3.2.3).

## Supplementary information

Supplementary Information
