# Supplementary material for: High pollutant exposure level of the largest European community of bottlenose dolphins in the English Channel
Source: Sci Rep. 2019 Sep 12;9:12521. doi: 10.1038/s41598-019-48485-7 (PMC6742642; doi:10.1038/s41598-019-48485-7)
Supplement: Supplementary file 1 — Supplementary Information [file 41598_2019_48485_MOESM1_ESM.docx]

**Supplementary Information**

**High pollutant exposure level of the largest European community of bottlenose dolphins in the English Channel**

Cyrielle Zanuttini^1^, François Gally^1^, Georges Scholl^2^, Jean-Pierre Thomé^3^, Gauthier Eppe^2^, Krishna Das^4^

^1^ Groupe d'Etude des Cétacés du Cotentin (GECC), Place des Justes, 50130, Cherbourg-Octeville, France

^2^ CART, UR MolSys B6c, University of Liège, 4000, Liège, Belgium

^3^ CART-LEAE, Freshwater and Oceanic sciences Unit of reSearch (FOCUS- CART-LEAE)^3^, B6C, University of Liège, Liège ,Belgium

^4^ Freshwater and Oceanic sciences Unit of reSearch (FOCUS-Oceanology), B6C, University of Liège, Liège ,Belgium

Corresponding author: [Krishna.das@uliege.be](mailto:Krishna.das@uliege.be)

**Table S1: Sampling description and pollutant investigations per individual**

| Individual | ∑6PCBs, ∑PBDEs, ∑DDTs, ∑HCHs, HCB  n=46 | PCDD/Fs, PCB-DL, ∑6PCBs, ∑PBDEs, ∑DDTs, ∑HCHs, HCB  n=12 | ∑Chlordane, Dieldrine, ∑Endosulfan  n=21 | T-Hg n=69 |
| --- | --- | --- | --- | --- |
| 1 |  |  |  | X |
| 2 | X |  |  | X |
| 3 | X |  |  | X |
| 4 |  |  |  | X |
| 5 | X |  | X | X |
| 6 |  |  |  | X |
| 7 | X |  |  | X |
| 8 | X |  | X | X |
| 9 |  |  |  | X |
| 10 |  | X |  | X |
| 11 |  |  | X | X |
| 12 |  |  |  | X |
| 13 |  |  |  | X |
| 14 | X |  | X | X |
| 15 |  | X |  | X |
| 16 | X |  | X | X |
| 17 | X |  |  | X |
| 18 | X |  |  | X |
| 19 |  | X | X | X |
| 20 | X |  | X | X |
| 21 | X |  |  | X |
| 22 |  |  | X | X |
| 23 |  |  | X | X |
| 24 | X |  |  |  |
| 25 | X |  |  |  |
| 26 | X |  |  | X |
| 27 |  |  |  | X |
| 28 | X |  |  | X |
| 29 | X |  |  |  |
| 30 | X |  | X | X |
| 31 |  | X |  | X |
| 32 | X |  |  | X |
| 33 | X |  |  | X |
| 34 |  |  |  | X |
| 35 |  |  |  | X |
| 36 | X |  |  | X |
| 37 | X |  | X | X |
| 38 | X |  |  | X |
| 39 |  |  |  | X |
| 40 |  |  |  | X |
| 41 |  |  |  | X |
| 42 |  |  |  | X |
| 43 | X |  |  | X |
| 44 |  |  |  | X |
| 45 | X |  | X | X |
| 46 | X |  |  | X |
| 47 |  | X |  | X |
| 48 | X |  |  | X |
| 49 | X |  |  |  |
| 50 |  | X |  | X |
| 51 | X |  |  | X |
| 52 | X |  |  | X |
| 53 |  | X |  | X |
| 54 |  |  | X |  |
| 55 | X |  |  | X |
| 56 |  |  | X |  |
| 57 | X |  |  | X |
| 58 | X |  |  | X |
| 59 | X |  | X | X |
| 60 | X |  | X | X |
| 61 |  | X |  | X |
| 62 |  | X |  |  |
| 63 | X |  |  |  |
| 64 |  | X | X |  |
| 65 | X |  | X | X |
| 66 |  |  | X | X |
| 67 | X |  |  |  |
| 68 |  |  |  | X |
| 69 | X |  |  | X |
| 70 |  |  | X | X |
| 71 | X |  |  | X |
| 72 | X |  |  | X |
| 73 | X |  |  | X |
| 74 |  |  | X |  |
| 75 | X |  |  | X |
| 76 |  |  |  | X |
| 77 | X |  |  |  |
| 78 | X |  |  |  |
| 79 |  | X |  | X |
| 80 |  | X |  | X |
| 81 | X |  |  | X |
| 82 | X |  |  | X |

Table S2: PCDD/Fs and DL-PCBs concentrations (pg.g^-1^ lipids) in male (n =9) and female (n=3) bottlenose dolphins from Normanno-Breton Gulf. Data are showed as mean concentrations, TEF (toxic equivalency factor) and TEQ (toxicity equivalent) (pg.g-1 lipids) and p-value (p<0.05). Significant differences for TEQ values are shown in bold.

| Congener | | Mean concentrations | | TEF | TEQ | | p-value |
| --- | --- | --- | --- | --- | --- | --- | --- |
|  | | *males* | *females* |  | *males* | *females* |  |
| PCDDs | |  |  |  |  |  |  |
|  | 2, 3, 7, 8 - TetraCDD | 0.18* | 0.18* | 1 | 0.18 | 0.18 | 1.00 |
|  | 1, 2, 3, 7, 8 – PentaCDD | 0.18* | 0.18* | 1 | 0.18 | 0.18 | 1.00 |
|  | 1, 2, 3, 4, 7, 8 – HexaCDD | 0.18* | 0.18* | 0.1 | 0.02 | 0.02 | 1.00 |
|  | 1, 2, 3, 6, 7, 8 – HexaCDD | 2.83 | 2.99 | 0.1 | 0.28 | 0.3 | 0.864 |
|  | 1, 2, 3, 7, 8, 9 - HexaCDD | 1.52 | 1.28 | 0.1 | 0.15 | 0.13 | 1.00 |
|  | 1, 2, 3, 4, 6, 7, 8 - HeptaCDD | 17.6 | 14.2 | 0.01 | 0.17 | 0.14 | 0.921 |
|  | OctaCDD (OCDD) | 37.8 | 32.5 | 0.0003 | 0.01 | 0.01 | 0.925 |
| PCDFs | |  |  |  |  |  |  |
|  | 2, 3, 7, 8 – TetraCDF | 1.62 | 10.44 | 0.1 | 0.16 | 1.04 | **0.038** |
|  | 1, 2, 3, 7, 8 – PentaCDF | 3.41 | 1.64 | 0.03 | 0.1 | 0.05 | **0.036** |
|  | 2, 3, 4, 7, 8 - PentaCDF | 4.85 | 3.65 | 0.3 | 1.45 | 1.09 | 0.373 |
|  | 1, 2, 3, 4, 7, 8 - HexaCDF | 12.3 | 3.82 | 0.1 | 1.23 | 0.38 | **0.009** |
|  | 1, 2, 3, 6, 7, 8 – HexaCDF | 1.00 | 1.03 | 0.1 | 0.1 | 0.1 | 0.565 |
|  | 1, 2, 3, 7, 8, 9 - HexaCDF | 206.8 | 41.8 | 0.1 | 20.7 | 4.18 | **0.009** |
|  | 2, 3, 4, 6, 7, 8 - HexaCDF | 17.3 | 6.81 | 0.1 | 1.73 | 0.68 | 0.100 |
|  | 1, 2, 3, 4, 6, 7, 8 - HeptaCDF | 17.8 | 18.6 | 0.01 | 0.18 | 0.18 | 0.576 |
|  | 1, 2, 3, 4, 7, 8, 9 - HeptaCDF | 0.91 | 0.18* | 0.01 | 0.009 | 0.002 | 0.700 |
|  | OctaCDF (OCDF) | 15.8 | 14.3 | 0.0003 | 0.005 | 0.004 | 1.00 |
| **∑ PCDD/Fs** | | **342** | **154** |  | **26.7** | **8.68** | **0.018** |
| Non-ortho PCBs | |  |  |  |  |  |  |
|  | PCB 77 | 2077 | 2202 | 0.0001 | 0.21 | 0.22 | 0.727 |
|  | PCB 81 | 273 | 274 | 0.0003 | 0.08 | 0.09 | 0.864 |
|  | PCB 126 | 1724 | 738 | 0.1 | 172 | 73.8 | 0.064 |
|  | PCB 169 | 335 | 337 | 0.03 | 10.0 | 10.1 | 0.482 |
| Mono-ortho PCBs | |  |  |  |  |  |  |
|  | PCB 105 | 1225632 | 416126 | 0.00003 | 36,8 | 12,48 | **0.009** |
|  | PCB 114 | 33266 | 9472 | 0.00003 | 1,00 | 0,28 | **0.009** |
|  | PCB 118 | 4499539 | 1500232 | 0.00003 | 135 | 45,0 | **0.009** |
|  | PCB 123 | 21055 | 7635 | 0.00003 | 0,63 | 0,23 | **0.009** |
|  | PCB 156 | 680270 | 231615 | 0.00003 | 20,4 | 6,95 | **0.009** |
|  | PCB 157 | 337779 | 87466 | 0.00003 | 10,1 | 2,62 | **0.009** |
|  | PCB 167 | 889783 | 257795 | 0.00003 | 26,7 | 7,73 | **0.009** |
|  | PCB 189 | 364275 | 102574 | 0.00003 | 10,9 | 3,08 | **0.009** |
| **∑ DL-PCBs (non-ortho PCBs and mono-ortho PCBs)** | | **8056008** | **2616467** |  | **424** | **162.6** | **0.009** |
| **∑ PCDD/Fs and DL-PCBs** | | **8056350** | **2616620** |  | **451** | **171** | **0.009** |

*Data determined on the basis of LOQ

# **Table S3: Total mercury concentrations (ng.g dw) in skin of bottlenose dolphins in literature (adapted from Damseaux et al. 2016, Supplementary data)**

|  | **Localisation** | **Date** | **n** | **Mean ± SD** | **median** | **Min-max** | **References** |
| --- | --- | --- | --- | --- | --- | --- | --- |
| **Biopsies** | **English Channel** | **2010-2012** | **69** | **9923 ± 4300** | **9319** | **2452-21,293** | **Present work** |
| Strandings or bycatch | Northeast Atlantic | 2001-2008 | 16 | 5700 ± 2900 |  | 2200-14,400 | ^1^ |
| Strandings | Corsica | 1997 | 1 | 15000 ± 900 |  |  | ^2^ |
| Biopsies | Florida –  LFK | 2008 | 10 | 2779 ± 2025 | 2941 | 294 – 5713 | ^3^ |
| Biopsies | Florida –  FCE | 2013 | 24 | 10916 ± 7507 | 9314 | 2221 – 29125 | ^3^ |
| Strandings | Florida - Indian River Lagoon | 2000-2008 | 15 | 8570 ± 7040 |  | 1520 - 22600 | ^4^ |
| Biopsies | Florida  Sarasota Bay | 2003-2005 | 54 | 2152 ± 1680 |  | 321.5 - 7685 | ^5^ |
| Strandings | South Carolina | 2000-2008 | 12 | 1810 ± 1810 |  | 169 - 5650 | ^4^ |
| Strandings or bycatch | Southeast Atlantic | 2003-2005 | 74 | 1700 ± 920 |  | 650 - 4900 | ^6^ |
|  |  |  |  |  |  |  |  |
| Captive animals | Baltimore National Aquarium | 2010 | 1 | 825 ± 380 |  |  | ^7^ |
| Live captures | Japan | 2007-2008 | 31 |  | 29960 |  | ^8^ |
| Biopsies | La Réunion | 2010-2011 | 32 |  | 2850 | 723 - 6520 | ^9^ |

# **A**

**B**

**Figure S1: Relationships between δ^13^C and δ ^15^N values and mercury concentration in skin of bottlenose dolphins from the Normanno-Breton Gulf**

# **Materials and methods**

## **Biopsy sample collection**

Biopsy samples from individual bottlenose dolphins were collected during boat surveys in the Normanno-Breton gulf from 2010 to 2012 (French ministry permit No. 09/115/DEROG).

Skin and blubber biopsies were obtained using a crossbow (Panzer Barnett 5), tips and arrows made by Finn Larsen (Danish Institute for Fisheries Research). Each individual biopsied was photo-identified using the natural marks on their dorsal fins. When a biopsy was successfully achieved, the skin was removed from the blubber and frozen at -20°C for mercury analysis, sex determination and stable isotope analysis. The remaining blubber was wrapped in aluminium foil inside a glass jar and stored at -80°C for POPs analysis. At the end, from a total of 82 bottlenose dolphins biopsied, we obtained for this study 79 blubber biopsies and 69 skin biopsies (Table S1, in Supporting information).

## **Gender identification and age class**

The gender of each individual biopsied was determined in a previous study ^10^. Briefly, DNA was extracted from the skin of the biopsy using NucleoSpin Tissue kits (Macherey-Nagel) and following the manufacturer’s protocol. Then, individuals were sexed by the amplification of the SRY and ZFX/ZFY gene fragments ^11^.

Photographs of individuals provided information about the dolphin’s status of maturity, adult or sub-adult, accordingly to morphological characteristics and dorsal fin marks ^12,13^. The three classes were defined as sub-adult, adult female and adult male.

## **Stable isotope analysis**

δ^13^C and δ^15^N values analysed previously were integrated in the present manuscript ^14^. Shortly, skin of each individual biopsied were dried at 45 °C for 48 h. After lipid extraction, δ^13^C and δ^15^N values were measured by a continuous flow mass spectrometer (Thermo Scientific, Delta V Advantage) coupled to an elemental analyser (Thermo Scientific, Flash EA 1112). Carbon and nitrogen isotopic ratios are expressed in the conventional δ notation (noted as δ^13^C and δ^15^N respectively) in parts per thousand (‰) using Vienna Pee Dee Belemnite for δ^13^C values and atmospheric N_2_ for δ^15^N values as international standard (IAEA, Vienna, Austria).

## **Persistent organic pollutants (POPs)**

Blubber samples were analysed for non-dioxins like (NDL) PCB congeners (no 28; 52; 101; 138; 153 and 180), dioxins-like (DL) PCBs congeners (no 77; 81; 105; 114; 118; 123; 126; 156; 157; 167; 169 and 189), 17 WHO PCDD/Fs, PBDEs (no 28; 47; 66; 85; 99; 100; 153; 154 and 183) and organochlorinated pesticides (ΣDDXs, cis-chlordane and trans-chlordane; α-Endosulfan, β-Endosulfan and Endosulfan-sulfate; dieldrin; HCB; α-HCH, β-HCH and γ-HCH) (Table S1).

The quantification of PCDD/Fs, DL-PCBs, NDL-PCBs, PBDEs, DDXs, HCB and HCHs was performed by the isotope dilution technique using ^13^C labelled analogues ^3,15^. Prior to the extraction, a known concentrations of ^13^C labelled version of the compounds of interest are added to each sample as internal standard. Then, the lipid fraction of the blubber was extracted via the Accelerated Solvent Extraction (ASE, Dionex 200, Thermo, USA) using dichloromethane as organic solvent. The extracted compounds were filtered with Na_2_SO_4_ and the solvent was evaporated until dryness under nitrogen flux. Lipids contents were then determined gravimetrically until constant weight. After, several clean up stages were performed using a multicolumn liquid-solid chromatography with an automated purification system, Power Prep^TM^ (FMS, Waltham, USA). This system removes major matrix interferences from the extracts and separates the mono-ortho fraction from the dioxin like compounds fraction. At the end of the purification process, recovery standards were added into the different fractions prior to GC-HRMS analysis using an Autospec Ultima High Res Mass Spectrometer coupled to an Agilent 6890 GC. The injection was carried out in splitless mode and the mass spectrometry via electron ionization (40 eV) using a selected ion-monitoring (SIM) mode. The samples having a lipid percentage lower than 2% were excluded from this study in order to express the concentrations on lipid weight (lw) basis. Thus, POP concentrations are expressed in ng.g^-1^ lw.

The toxic equivalent quantity (TEQ) for PCDD/Fs and DL-PCB was calculated to assess their toxic potency in the bottlenose dolphin’s blubber. Thus, the concentrations of each dioxin like compound was multiplied by their corresponding toxic equivalency factor (TEF) recommended by the World Health Organization ^16^. The TEQ is expressed in pg WHO-TEQ.g lw.

21 blubber biopsies were selected for the analysis of chlorinated pesticides Cis-Chlordane, Trans-Chlordane, α-Endosulfan, β-Endosulfan, Endosulfan-sulfate; and Dieldrin. Pesticides extraction and clean-up were performed by an appropriate method to preserve the acid sensitive organochlorine pesticides. The lipid fraction of the blubber was extracted by means of an Accelerated Solvent Extraction (ASE, Dionex 200, Thermo, USA) on 100 to 200 mg wet weight of blubber using a mixture of hexane:acetone (50:50; V:V) at 125°C and 1.304.10^7^ Pa. Prior to the extraction, 50µL of a hexanic solution of PCB congener 112 (Dr. Ehrenstorfer®, Augsburg, Germany) at 100 pg µL^-1^was added to the samples as a surrogate internal standard used to measure the recovery efficiency. The fat content was determined gravimetrically after solvent evaporation with a TurboVap LV concentration Evaporator workstation (Zymark TurboVap®LV, Charlotte, USA). The lipid extract was resuspended in 1 mL n-Hexane . 1 mL of a solution of Acetonitrile: Dichloromethane (95:5; V:V) was added to the lipid hexanic extract. After mixing, the acetonitrile:dichloromethane phase was recovered and placed at the top of the C_18_ microcartridge (SUPELCO, Sigma-Aldrich, Overijse, Belgium).. The compounds of interest were eluted with 5 mL of acetonitrile: dichloromethane (95:5; V:V) and the extract was evaporated under a gentle stream of nitrogen to a final volume of 500 µL. 1 mL of n-Hexane was added to the extract which was placed at the top of the Envi-Florisil microcartridge (SUPELCO, Sigma-Aldrich, Overijse, Belgium). The organochlorine pesticides analysed by this method were eluted with 10 mL of hexane:acetone (90:10; V:V). Five µL of nonane were added as a keeper to the purified extract. Each extract was evaporated under a gentle stream of nitrogen till just the keeper remains in the vial. The final extract was reconstituted with 45 µL of n-hexane and 50 µL of PCB80 (100 pg µL^-1^ in hexane) as injection volume internal standard (Dr. Erhenstorfer® GmbH, Augsburg, Germany). This compound such as PCB112 were never detected in such samples from these locations during pre-test analysis. The quantification was performed by means of the internal standard method. A calibration curve (1.5 - 250 pg µL^-1^) was established for each compound of interest. The final extracts were analysed by high resolution gas chromatograph coupled to an ion trap mass spectrometer (Trace GC Ultra and ITQ 1100 from ThermoQuest) using a 30 m x 0.25 mm (0.25 mm film) DB5 ms capillary column (J&W Scientific, USA). The chromatographic conditions were described elsewhere ^17^. The transfer line temperature was kept at 290°C and the ion trap temperature was set at 250°C. The electron ionization (EI) was performed at 70 eV and the ion trap was operating in MS/MS mode. The quality control (QC) was commercial pork fat, free of the compounds of interest. The pork fat was spiked with nominal concentrations of organochlorine pesticides of 5 ng g^-1^ lipid weight forming the QC. The pesticide concentrations in each sample and in the QC were corrected for initial sample weight, and the percentage recovery of the surrogate PCB 112. Recovery rates ranged between 83% ± 12% and 116% ± 5% according to the pesticide in the QC. These values are in good agreement with the requirements of SANCO: 70 to 130 % of recovery (SANCO, 2014). The limit of detection (LOD) was 0.02 ng.g^-1^ lipid weight and the measured limit of quantification (LOQ) determined with pesticide spiked pork fat was 0.07ng.g^-1^ lipid weight.

### **Total mercury (T-Hg)**

Total mercury analysis were performed on skin samples from 69 bottlenose dolphins as previously described ^3^. Briefly, T-Hg concentrations were measured by atomic absorption spectroscopy (AAS, Direct Mercury Analyzer DMA-80 Milestone) according to the US EPA standard method 7473. T-Hg concentrations are expressed in ng.g^-1^ dry weight (dw).

## **Statistical analysis**

Non-parametric statistics were used because the assumptions of normality (Shapiro test) and homoscedasticity (Bartlett test) of the data were not met, even after a log-transformation. Significant differences between contaminant concentrations in sex and marking level were assessed using Mann-Whitney *U* test for comparison of two categories and Kruskal-Wallis test for greater than two categories. If a significant difference was found, the post hoc Nemenyi’s test was performed to identify which group is significantly different. Spearman rank correlation tests were performed to examine the potential linear association between contaminants and stable isotopes. A statistical significance level of 0.05 was applied for all tests. Statistical analyses were conducted with R studio software (version 3.2.3).

**References**

1. Aubail, A. *et al.* Use of skin and blubber tissues of small cetaceans to assess the trace element content of internal organs. *Mar. Pollut. Bull.* **76,** 158–169 (2013).

2. Frodello, J. P., Viale, D. & Marchand, B. Metal concentrations in the milk and tissues of a nursing Tursiops truncatus female. *Mar. Pollut. Bull.* **44,** 551–576 (2002).

3. Damseaux, F. *et al.* Spatial variation in the accumulation of POPs and mercury in bottlenose dolphins of the Lower Florida Keys and the coastal Everglades (South Florida). *Environ. Pollut.* **220,** 577–587 (2017).

4. Stavros, H.-C. W. *et al.* Correlation and toxicological inference of trace elements in tissues from stranded and free-ranging bottlenose dolphins (Tursiops truncatus). *Chemosphere* **82,** 1649–1661 (2011).

5. Woshner, V. *et al.* Mercury and selenium in blood and epidermis of bottlenose dolphins (Tursiops truncatus) from sarasota bay, FL: Interaction and relevance to life history and hematologic parameters. *Ecohealth* **5,** 360–370 (2008).

6. Stavros, H.-C. W., Bossart, G. D., Hulsey, T. C. & Fair, P. A. Trace element concentrations in skin of free-ranging bottlenose dolphins (Tursiops truncatus) from the southeast Atlantic coast. *Sci. Total Environ.* **388,** 300–315 (2007).

7. Hong, Y. S., Hunter, S., Clayton, L. a, Rifkin, E. & Bouwer, E. J. Assessment of mercury and selenium concentrations in captive bottlenose dolphin’s (Tursiops truncatus) diet fish, blood, and tissue. *Sci. Total Environ.* **414,** 220–6 (2012).

8. Sakamoto, M. *et al.* Mercury speciation and selenium in toothed-whale muscles. *Environ. Res.* **143,** 55–61 (2015).

9. Dirtu, A. C. *et al.* Contrasted accumulation patterns of persistent organic pollutants and mercury in sympatric tropical dolphins from the south-western Indian Ocean. *Environ. Res.* **146,** 263–273 (2016).

10. Louis, M. *et al.* Habitat-driven population structure of bottlenose dolphins, Tursiops truncatus, in the North-East Atlantic. *Mol. Ecol.* **23,** 857–874 (2014).

11. Rosel, P. E. PCR-based sex determination in Odontocete cetaceans. *Conserv. Genet.* **4,** 647–649 (2003).

12. Wilson, B., Hammond, P. S. & Thompson, P. M. Estimating size and assessing trends in a coastal bottlenose dolphin population. *Ecol. Appl.* **9,** 288–300 (1999).

13. Gibson, Q. A. & Mann, J. The size, composition and function of wild bottlenose dolphin (Tursiops sp.) mother–calf groups in Shark Bay, Australia. *Anim. Behav.* **76,** 389–405 (2008).

14. Louis, M. *et al.* Ecological opportunities and specializations shaped genetic divergence in a highly mobile marine top predator. *Proc. R. Soc. B Biol. Sci.* **281,** (2014).

15. Focant, J. F., Eppe, G., Pirard, C. & De Pauw, E. Fast clean-up for polychlorinated dibenzo-p-dioxins, dibenzofurans and coplanar polychlorinated biphenyls analysis of high-fat-content biological samples. *J. Chromatogr. A* **925,** 207–221 (2001).

16. Van den Berg, M. *et al.* The 2005 World Health Organization reevaluation of human and mammalian toxic equivalency factors for dioxins and dioxin-like compounds. *Toxicol. Sci.* **93,** 223–241 (2006).

17. Debier, C. *et al.* Dynamics of PCB transfer from mother to pup during lactation in UK grey seals Halichoerus grypus: differences in PCB profile between compartments of transfer and changes during the lactation period. *Mar. Ecol. Ser.* **247,** 249–256 (2003).
